# Peroxiredoxin 5 Silencing Sensitizes Dopaminergic Neuronal Cells to Rotenone via DNA Damage-Triggered ATM/p53/PUMA Signaling-Mediated Apoptosis

**DOI:** 10.3390/cells9010022

**Published:** 2019-12-19

**Authors:** Mei-Jen Wang, Hsin-Yi Huang, Tsung-Lang Chiu, Hui-Fen Chang, Hsin-Rong Wu

**Affiliations:** 1 Department of Medical Research, Hualien Tzu Chi Hospital, Buddhist Tzu Chi Medical Foundation, Hualien 970, Taiwan; hyhuang@tzuchi.com.tw (H.-Y.H.); check172@tzuchi.com.tw (H.-F.C.); rong3016@gmail.com (H.-R.W.); 2 Division of Neurosurgery, Neuro-Medical Scientific Center, Hualien Tzu Chi Hospital, Buddhist Tzu Chi Medical Foundation, Hualien 970, Taiwan; poluschiou@gmail.com; 3 School of Medicine, Tzu Chi University, Hualien 970, Taiwan

**Keywords:** Parkinson’s disease, dopaminergic neurons, peroxiredoxin 5, rotenone, P53, PUMA, DNA damage, γ-H2AX, ATM

## Abstract

Peroxiredoxins (Prxs) are a family of thioredoxin peroxidases. Accumulating evidence suggests that changes in the expression of Prxs may be involved in neurodegenerative diseases pathology. However, the expression and function of Prxs in Parkinson’s disease (PD) remains unclear. Here, we showed that Prx5 was the most downregulated of the six Prx subtypes in dopaminergic (DA) neurons in rotenone-induced cellular and rat models of PD, suggesting possible roles in regulating their survival. Depletion of Prx5 sensitized SH-SY5Y DA neuronal cells to rotenone-induced apoptosis. The extent of mitochondrial membrane potential collapse, cytochrome c release, and caspase activation was increased by Prx5 loss. Furthermore, Prx5 knockdown enhanced the induction of PUMA by rotenone through a p53-dependent mechanism. Using RNA interference approaches, we demonstrated that the p53/PUMA signaling was essential for Prx5 silencing-exacerbated mitochondria-driven apoptosis. Additionally, downregulation of Prx5 augmented rotenone-induced DNA damage manifested as induction of phosphorylated histone H2AX (γ-H2AX) and activation of ataxia telangiectasia mutated (ATM) kinase. The pharmacological inactivation of ATM revealed that ATM was integral to p53 activation by DNA damage. These findings provided a novel link between Prx5 and DNA damage-triggered ATM/p53/PUMA signaling in a rotenone-induced PD model. Thus, Prx5 might play an important role in protection against rotenone-induced DA neurodegeneration.

## 1. Introduction

The progressive degeneration of dopaminergic (DA) neurons in the substantia nigra (SN) pars compacta and depletion of striatal dopamine are hallmarks of Parkinson’s disease (PD). Its cardinal clinical symptoms include bradykinesia, rigidity, resting tremor, and disturbances in balance. A combination of environmental, genetic, and epigenetic factors has been thought to contribute to the etiology of PD [1,2]. Although the molecular pathogenesis of PD is not thoroughly understood, there is mounting evidence to suggest that mitochondrial dysfunction, oxidative stress, impairment of the ubiquitin-proteasome system, and autophagy-lysosome pathway may be involved in the pathogenesis of PD [3,4]. Recently, DNA damage is hypothesized to constitute a unifying component across different neurodegenerative diseases [5]. Increased DNA strand breaks and oxidative DNA damages have been found in the SN and peripheral blood of PD patients [6,7,8], suggesting that DNA damage may compromise genomic integrity leading to the onset of PD [9].

Peroxiredoxins (Prxs) represent a superfamily of thiol-dependent antioxidant enzymes that participate directly in eliminating H_2_O_2_ and regulate the transduction of various cellular signals by modulating redox signaling [10,11]. In mammals, six isoforms of Prxs have been identified, comprising typical 2-Cys Prxs (Prx1-4), atypical 2-Cys Prxs (Prx5), and 1-Cys Prxs (Prx6). Proposed functions of Prxs include regulation of cellular proliferation, differentiation, immune responses, and apoptosis [12]. Prx5 is broadly localized in the cytosol, mitochondria, peroxisome, and nucleus, and performs specific functions according to its subcellular localization [13]. Initially, Prx5 was described as a DNA-binding protein able to suppress RNA polymerase III-dependent transcription of Alu retroposons in vitro [14]. The cytoprotective antioxidant function of Prx5 has been investigated after human Prx5 was characterized as a peroxidase but also because of the existence of this enzyme in peroxisomes and mitochondria, the two major places of reactive oxygen species (ROS)/reactive nitrogen species generation in cells [13]. Furthermore, a previous study reported that the prevention of etoposide-induced DNA damage by Prx5 in human cells was not attributed to the antioxidant activity but depended on another functional activity of this protein [15]. Prx5 plays a role in protecting various cell types, including neurons [13,16,17,18]. Exogenous administration of recombinant human Prx5 was demonstrated to provide neuroprotection against excitotoxic stress induced by ibotenate in the mouse brain [19]. Prx5 also exerts protective effect against 1-methyl-4-phenylpyridinium (MPP^+^)-induced neurodegeneration [16]. In addition, recent work showed that Prx5 prevented amyloid-β oligomer-caused neuronal cell death [17,18].

An increasing body of evidence suggests that modifications or changes in the expression of Prx subtypes may be implicated in the pathogenesis of neurodegenerative disorders, including PD [18,20,21,22,23]. Our investigation demonstrated that Prx5 was the most downregulated of the six Prx subtypes in DA neurons following exposure to rotenone in vitro and in vivo. Despite the evidence for the role of Prx5 in protecting neurons [16,17,18], the biological function of Prx5 in regulating DA neuronal cell death associated with PD has not been completely elucidated. Based on the epidemiological studies, environmental risk factors, such as pesticide exposure, can be a major cause for PD. Thus, in the present study, a rotenone-induced cellular model of PD was used to investigate the effect of Prx5 loss on DA neuronal survival and to delineate the molecular mechanisms underlying this effect.

## 2. Materials and Methods

### 2.1. Cell Cultures

Primary rat ventral mesencephalic cultures were prepared following our published protocol [24]. Briefly, ventral mesencephalic tissues were dissected from embryonic day 14.5 Sprague-Dawley rats and dissociated enzymatically (0.1% trypsin-EDTA) and mechanically. Dissociated rat ventral mesencephalic cells were resuspended in neurobasal medium containing 0.5 mM glutamine, 25 μM glutamate, 2% B27 supplement (Invitrogen, Carlsbad, CA, USA), 100 U/mL penicillin, and 100 μg/mL streptomycin and seeded to 24-well (8 × 10^5^/well) culture plates pre-coated with poly-*d*-lysine (20 μg/mL). Cultures were maintained at 37 °C in a humidified atmosphere of 5% CO_2_ and 95% air. Four days later, the medium was changed to fresh neurobasal/B27 medium without glutamate. The mesencephalic neuron-enriched cultures were incubated for another three days prior to the experiments.

Human DA neuroblastoma SH-SY5Y cells were grown in 1:1 mixture of modified Eagle’s medium (MEM) and Ham’s F12 nutrient mix, 10% fetal bovine serum (FBS), 1% non-essential amino acids, 2 mM l-glutamine, 100 U/mL penicillin, and 100 μg/mL streptomycin and incubated at 37 °C in a humidified atmosphere of 5% CO_2_.

### 2.2. Animals and Treatment

All animal experimental protocols were approved by the Institutional Animal Care and Use Committee of the Buddhist Tzu Chi General Hospital in accordance with guidelines set by the National Institutes of Health Guide for the Care and Use of Laboratory Animals. Animals were housed in an animal facility under a 12 h light/dark cycle with food and water *ad libitum*. The procedure of animal treatment was performed according to the previous study [25]. Male Sprague-Dawley rats (276–300 g) were randomly divided into control and rotenone groups and underwent 28 days of treatments. Rats received vehicle solution (DMSO + corn oil) or rotenone (1.5 mg/kg/day) (5 times a week) for 4 weeks. Rotenone was dissolved in DMSO and diluted in sterile corn oil (2% final DMSO concentration and 98% final corn oil concentration). Rotenone and the vehicle solution were given by subcutaneous injection. All drugs were administered at 1 mL/kg/day. After the administration of rotenone, the rats were killed by decapitation following anesthesia. Brains were quickly removed, and the whole SN and striatum tissues were homogenized in T-PER^®®®®^ Tissue Protein Extraction Reagent (Pierce, Rockford, IL, USA). The homogenates were stored at −80 °C until use.

### 2.3. Lentiviral Transduction

For the silencing of target genes in SH-SY5Y cells, cells were infected with lentivirus. The lentiviral vectors, expressing short hairpin interfering RNA (shRNA) against human Prx5, PUMA, and p53, were obtained from National Core Facility for Manipulation of Gene Function/Genomic Research Center, Academia Sinica (Taipei, Taiwan). The control shRNA sequence that does not have any target genes in mammalian cells was also purchased. The target sequences of shRNA are as follows: Prx5, 5′-CCTCCCTATCTCACCTGCCCA-3′; PUMA, 5′-GTACAATCTCATCATGGGACT-3′; p53, 5′-CACCATCCACTACAACTACAT-3′. The production of the virus was performed using a protocol described previously [26]. For lentiviral transduction, SH-SY5Y cells were incubated with the lentiviral particles (multiplicity of infection, 20 for shPrx5 or 2.5 for shp53 and shPUMA) containing 4 μg/mL polybrene for 24 h, followed by medium change. All infected cells were selected with puromycin (2 μg/mL) or with both puromycin and blasticidin (2 μg/mL) until the stable knockdown of investigated genes. For experiments, cultures were washed with serum-free medium, and the medium was replaced with lower serum (0.5% FBS) medium before exposure to rotenone until they were harvested for various assays.

### 2.4. Sirna and Prx5-Expressing Plasmid Transfection

Rat Accell^TM^ SMARTpool Prx5 (item^#^: E-096041-00-0005) siRNA was obtained from Dharmacon (Thermo Scientific, Lafayette, CO, USA). Nonspecific siRNA was used as a negative control. Mesencephalic neurons were seeded in 24-well plates for 3 days prior to transfection. siRNA duplexes were transfected into mesencephalic neurons using the Accell^TM^ siRNA delivery system according to the manufacture’s instruction. After 3 days of transfection, cultures were exposed to 100 nM rotenone for 24 h. DA neuron degeneration was examined following immunocytochemistry. For Prx5 knock-in experiments, a vector carrying cDNA of human Prx5 (catalog no.: RC210709L3) or the empty vector (catalog no.: PS100092) (both from OriGene Technologies, Rockville, MD, USA) were transfected into Prx5-depleted cells using Lipofectamine^TM^ 3000 Reagent (Invitrogen) according to the protocol of the manufacturer. At 30 h after transfection, cells were treated with 10 μM rotenone for another 36 h. Cell viability was determined by MTT assay.

### 2.5. Immunocytochemistry

The degeneration of DA neurons was assessed by counting the number of tyrosine hydroxylase (TH)-positive cells following immunostaining in mesencephalic cultures. Briefly, mesencephalic neuron-enriched cultures were fixed with 4% paraformaldehyde, followed by blocking with PBS containing 0.3% Triton X-100 and 2% bovine serum albumin (BSA) for 1 h at room temperature. After blocking, cells were incubated overnight at room temperature with primary antibodies. Primary antibodies were mouse anti-tyrosine hydroxylase (TH), rabbit anti-TH (both from Millipore, Billerica, MA, USA), and rabbit anti-Prx5 (Abcam, Cambridge, MA, USA). For the detection of DA neurons, the bound anti-TH antibody was visualized by incubation with an appropriate biotinylated secondary antibody followed by the Vectastain avidin-biotin-peroxidase (ABC) reagents (Vector Laboratories, Burlingame, CA, USA) and color development with 3,3′-diaminobenzidine. The numbers of TH-positive neurons were counted in the entire surface area of a culture well. For fluorescent double-labeling experiments, cells were incubated for 1 h at room temperature with the secondary antibodies conjugated to the Alexa Fluor-488 or -594 (all from Jackson Immuno Research, West Grove, PA, USA). For immunodetection of phospho-histone H2AX (γ-H2AX) in SH-SY5Y cells, rotenone-treated cells were fixed, permeabilized, and incubated with rabbit anti-γ-H2AX (Cell Signaling Technology, Beverly, MA, USA). After washing, cells were incubated with Alexa Fluor-488-conjugated secondary antibody. Samples were counterstained with 4’,6-Diamidino-2-phenylindole (DAPI) (1 μg/mL) and mounted with 50% glycerol in PBS. Microscopic observations were done with a Zeiss LSM 510 META confocal imaging system (Carl Zeiss, Oberkochen, Germany).

### 2.6. Immunohistochemistry

During deep anesthesia, rats were perfused through the left ventricle with saline followed by 4% paraformaldehyde. Brains were removed and then post-fixed in 4% paraformaldehyde at room temperature for 2 h, cryoprotected in 30% (*w/v*) sucrose (4 °C), frozen, and stored at −80 °C. Coronal sections containing the striatum (40 μm) and the SN (20 μm) were cut by a Leica freezing microtome (Solms, Wetzlar, Germany). After blocking with 2% BSA in PBS, sections of striatum and SN were incubated overnight with a mouse anti-TH antibody. The antibody was detected using an ABC Elite kit with a 3,3′-diaminobenzidine solution in the absence (for SN sections) or presence of 15 mg/mL ammonium nickel sulfate (for striatum sections). For immunofluorescent double-labeling, the sections of SN were incubated with mouse anti-TH antibody and rabbit anti-Prx5 antibody. After washing, secondary antibodies were applied to sections for 1 h. Sections were then washed, mounted on slides, coverslipped with Vectashield mounting media (Vector Laboratories, Burlingame, CA, USA), and examined with a confocal microscope.

### 2.7. Cell Viability Assay

Modified 3-(4,5-dimethylthiozol-2-yl)-2,5-diphenyl-tetrazolium bromide (MTT) rapid colorimetric assay was used to evaluate the viability of cultured cells. Briefly, cells were cultured in 24-well plates and treated with various concentrations of rotenone for the indicated periods. The MTT (0.3 mg/mL in DMEM) was added to the cultures and incubated for 1 h at 37 °C. The absorbance was measured at 550 nm using a microplate ELISA reader (Molecular Devices, Silicon Valley, CA, USA). The optical density of the result in MTT assay was directly proportional to the number of viable cells.

### 2.8. Apoptosis Assay

For apoptotic cell determination, cells were stained with Annexin V and propidium iodide (PI) using FITC Annexin V/Dead Cell Apoptosis kit (Invitrogen) or Annexin V-allophycocyanin (APC) (eBioscience^TM^ Hu Annexin V-APC Recomb Protein, Invitrogen) according to the manufacturer’s protocol. After treatment, cells were harvested and washed once with cold PBS. Cells were then stained in a 100 μL of binding buffer containing Annexin V-FITC or Annexin V-APC and PI for 15 min in the dark at room temperature. The reaction was stopped by adding 400 μL of binding buffer, and cells were immediately analyzed using the FACSCalibur flow cytometer (BD Bioscience, San Diego, CA, USA). The Annexin V^+^/PI^−^ and Annexin V^+^/PI^+^ cells are defined as early apoptotic and late apoptotic cells, respectively.

Apoptosis was also assessed by terminal deoxynucleotidyl transferase-mediated dUTP nick end labeling (TUNEL) apoptosis detection kit (In Situ Cell Death Detection kit, Roche, Mannheim, Germany) according to the manufacturer’s instructions. After fixing and permeabilizing, cells were incubated with TUNEL reaction mixture for 1 h at 37 °C. Cells were rinsed with PBS and subsequently counterstained with DAPI. For the quantification of apoptotic cells, the preparation was visualized under a Zeiss Axiovert 200 M fluorescent microscope. The percentage of apoptotic cells was quantified by counting at least 3000 cells under each experimental condition.

### 2.9. Flow Cytometry

For the evaluation of changes in the mitochondrial membrane potential (Δψ*m*), cells were stained with 5,5′,6,6′-tetrachloro-1,1′,3,3′-tetraethyl-benzimidazolylcarbocyanine iodide (JC-1, Molecular Probes, Eugene, OR, USA). The shifts in both red and green fluorescence emissions of JC-1 were measured. Intracellular ROS and mitochondrial superoxide generation were assessed using carboxy-H_2_DCFDA and MitoSOX, respectively (Molecular Probes). Briefly, cells were treated with rotenone for the indicated times. After treatment, cells were loaded with 10 μg/mL JC-1, 10 μM carboxy-H_2_DCFDA, or 2 μM MitoSOX in the dark at 37 °C for 30 min. The cells were then washed with PBS, harvested by trypsinization, and analyzed with flow cytometry.

### 2.10. Real-Time RT-PCR Analysis

Total RNA was extracted from SH-SY5Y cells with TRIzol^®®®®^ reagent (Invitrogen). One-step real-time RT-PCR analysis was performed to determine the expression of genes (Power SYBR^®®®®^ Green RNA-to-C_T_^TM^ 1-step kit, Applied Biosystems, Foster City, CA, USA). The primer sequences are as follows: PUMA, 5′-GAC CTC AAC GCA CAG TAC GAG-3′ and 5′-AGG AGT CCC ATG ATG AGA TTG T-3′; p53, 5′-GCC AAA GAA GAA ACC ACT GGA TG-3′ and 5′-TGA GTT CCA AGG CCT CAT TCA G-3′; β-actin, 5′-CTG GGT ATG GAA TCT TGC-3′ and 5′-GTT GGC GTA CAG GTC TTT-3′. The threshold cycle (C_t_) value for each test gene was normalized to the C_t_ value for the β-actin control from the same RNA preparations. The ratio of transcription of each gene was calculated as 2^–(^^ΔCt)^, where ΔC_t_ is the difference C_t (test gene)_-C_t (β-actin)_.

### 2.11. Preparation of Whole-Cell Extracts and Subcellular Fractionations

After drug treatment, the mesencephalic neurons or SH-SY5Y cells were washed twice with ice-cold PBS and lysed in M-PER^®®®®^ mammalian protein extraction reagent (Pierce) containing 5 mM sodium orthovanadate and protease inhibitor cocktail (Roche). After incubation for 5 min, cell lysates were centrifuged, and the supernatants were collected. Cytosolic and mitochondria-enriched fractions were obtained from SH-SY5Y cells. Briefly, the cell suspension was mixed with an equal volume of digitonin (1 mg/mL) in PBS, incubated at 25 °C for 5 min, and then kept on ice for another 5 min. The mixtures were centrifuged at 15,000× g for 2 min, and the supernatant served as the cytosolic fraction. The pellet containing mitochondria was dissolved in 0.5% Triton X-100 in PBS, the insoluble material was removed by centrifugation, and the supernatant served as the mitochondria fraction. Nuclear extracts were prepared by using the NE-PER^®®®®^ nuclear and cytoplasmic extraction reagents (Pierce) as per the manufacturer’s instructions. The protein concentration of samples was determined by Bradford assay (Bio-Rad, Hercules, CA, USA), and aliquots were stored at −80 °C.

### 2.12. Western Blotting

A total of 10–30 μg of protein sample was separated on 6%–12% sodium dodecyl sulfate-polyacrylamide gel (SDS-PAGE) and transferred to immobilon polyvinylidene difluoride (PVDF) membranes (Millipore). The membranes were incubated in Tris-buffered saline (TBST, 0.1 M Tris/HCl, pH 7.4, 0.9% NaCl, 0.1% Tween 20) supplemented with 5% dry skim milk for 1 h to block nonspecific binding. After rinsing with TBST buffer, the membranes were incubated with primary antibodies overnight at 4 °C. Primary antibodies were rabbit anti-cytochrome C, rabbit anti-COX IV, rabbit anti-PUMA, rabbit anti-p-p53 (ser15), rabbit anti-p53, rabbit anti-cleaved caspase-3, rabbit anti-cleaved caspase-9, rabbit anti-cleaved PARP (poly (ADP-ribose) polymerase), rabbit anti-p-ATR (ataxia telangiectasia and Rad3-related) (Ser428), mouse anti-p-ATM (ataxia telangiectasia mutated) (Ser1981), rabbit anti-p-Histone H2AX (Ser139), rabbit anti-ATM, rabbit anti-ATR, mouse anti-HDAC1 (histone deacetylase 1) (all from Cell Signaling Technology), rabbit anti-TH (Millipore), rabbit anti-Prx1-6, mouse anti-GAPDH (all from Abcam), and mouse anti-β-actin (Sigma-Aldrich, St. Louis, MO, USA). The membranes were washed three times with TBST followed by incubation with appropriate horseradish peroxidase-conjugated secondary antibodies. After the removal of excess antibodies by TBST washing, specific binding was detected by using an ECL chemiluminescence detection system (PerkinElmer, Boston, MA, USA) according to the manufacturer’s instructions. The intensity of the bands was quantified with a GS-800 calibrated densitometer (Bio-Rad) and calculated as the optical density X area of bands.

### 2.13. Caspase Activity Assay

SH-SY5Y cells were harvested in caspase lysis buffer (PathScan^®®®®^ sandwich ELISA lysis buffer, Cell Signaling Technology). The caspase-3-like activity was assayed using the Caspase-3 Activity Assay kit (Cell Signaling Technology) according to the manufacturer’s instructions. Briefly, 50 μL of lysates were added to 150 μL of assay buffer containing 1 mg/mL *N*-acetyl-Asp-Glu-Val-Asp-(7-amino-4-methylcoumarin) (Ac-DEVD-AMC), a fluorogenic substrate cleaved by caspase-3. Accumulation of AMC fluorescence was monitored over 180 min using an FLx800 fluorescent plate reader (BioTek, Winooski, VT, USA) at excitation and emission wavelengths of 380 and 460 nm, respectively. Fluorescence of each sample reading at time 0 h was subtracted from the determined value. AMC fluorescence (Relative fluorescent units, RFU) of each sample was normalized to the protein content of the extracts. Caspase-3-like activity from the untreated sample was arbitrarily set at 1 for the calculation of fold induction.

### 2.14. Statistical Analysis

All data are expressed as mean ± SEM. Data were analyzed by one-way ANOVA, followed by Scheffe’s test. For paired analyses, a *t*-test was used. A *p*-value of less than 0.05 was considered statistically significant.

## 3. Results

### 3.1. Decrease of Prx5 Expression in DA Neurons in Rotenone-Induced Cellular and Rat Models of PD

**Figure 1:**
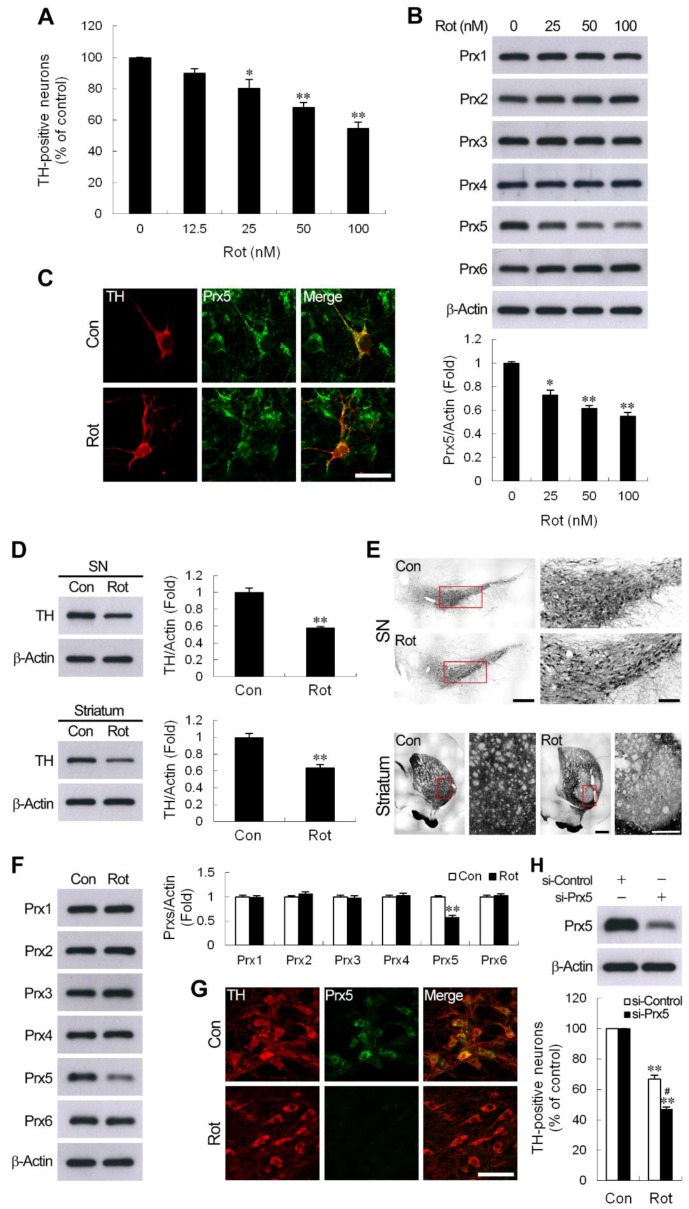
Rotenone reduced peroxiredoxin 5 (Prx5) expression in dopaminergic (DA) neurons in vitro and in vivo. (**A**,**B**) Mesencephalic neuron-enriched cultures were treated with various concentrations of rotenone (Rot) for 24 h. Degeneration of DA neurons was evaluated by counting the number of tyrosine hydroxylase (TH)-positive neurons (**A**). Whole-cell lysates were prepared and subjected to western blotting using Abs specific for Prx1-6 (**B**). Data are presented as mean ± SEM for four independent experiments. * *p* < 0.05; ** *p* < 0.01 compared with control. (**C**) Immunodetection of Prx5 in cultured DA neurons. Mesencephalic neuron-enriched cultures were exposed to 100 nM rotenone for 24 h, then fixed and double immunostained using anti-TH antibody and anti-Prx5 antibody. Scale bar = 25 μm. (**D**,**E**) Rotenone administration decreased TH expression in the rat substantia nigra (SN) and striatum. Rats were subcutaneously injected with rotenone or vehicle of the same volume for 4 weeks, as described in “Materials and Methods”. The protein extracts from SN and striatum tissues were processed for immunoblotting against TH (**D**) ** *p* < 0.01 compared with vehicle-injection controls; n = 5–6/group. (**E**) Representative immunohistochemical images of brain sections showing TH immunoreactive neurons and fibers in the SN and striatum, respectively. Low and high magnification views are shown in the left and right panels, respectively. Scale bar = 500 μm in the left panel of SN and striatum; 150 μm and 100 μm in the right panel of SN and striatum, respectively. (**F**,**G**) Rats received rotenone, as described in (**D)**. Protein levels of Prx1-6 in SN extracts were assessed by western blotting (**F**), ** *p* < 0.01 compared with vehicle-injection controls; n = 5–6/group. (**G**) Brain sections were subjected to double-label immunofluorescent staining for TH and Prx5 in the SN at 28 days after rotenone injection. Scale bar = 50 μm. (**H**) Prx5 knockdown increased rotenone-induced DA neuronal death. Mesencephalic neuron-enriched cultures from day 3 in vitro were transfected with control or Prx5 siRNA for 72 h. Cells were exposed to 100 nM rotenone for 24 h, and then viable DA neurons immunostained with TH were counted. Data are presented as mean ± SEM for four independent experiments. ** *p* < 0.01 compared with respective control. ^#^ *p* < 0.05 compared with rotenone-treated control siRNA transfected cells.

Rotenone, a mitochondrial complex I inhibitor, has been successfully used to induce a model of PD [27]. In the present study, the death of DA neurons in mesencephalic neuron-enriched cultures exposed to rotenone was determined by measuring the TH-positive cells counted. We observed that treatment of cultures with rotenone significantly increased the loss of TH-positive neurons in a dose-dependent manner (Figure 1A). Previous studies demonstrated that Prxs exerted protective effects against 6-hydroxydopamine (6-OHDA), or 1-methyl-4-phenyl-1,2,3,6,-tetrahydro-pyridine (MPTP)/MPP^+^-induced DA neuronal death in vitro and in vivo [16,22,23,28]. To examine which Prxs may affect the death of midbrain DA neurons resulted from the rotenone challenge, we first assessed the protein levels of the six Prx subtypes in rotenone-treated mesencephalic neuron-enriched cultures. As shown in Figure 1B, expression of Prx5 was significantly reduced, while that of other Prxs was not obviously changed in mesencephalic neurons exposed to rotenone. Furthermore, a rotenone-caused decrease of Prx5 was also observed at the cellular level since Prx5-immunoreactivity was reduced in DA neurons (Figure 1C). As mentioned above, rotenone treatment resulted in a reduction of the expression of Prx5 in cultured DA neurons. A rotenone-induced rat model of PD was used to confirm these results in vivo. The results showed that after rotenone administration, the expression of TH in the SN and striatum was significantly reduced (Figure 1D). Moreover, rotenone caused a loss of TH-positive neurons in the SN, and TH-positive fibers in the striatum were also observed (Figure 1E). In this rat PD model, we found that Prx5 was the most downregulated of the six Prx subtypes in the SN and DA neurons detected by western blotting and immunohistochemical staining, respectively (Figure 1F,G). These results suggested that Prx5 expression might play a role against rotenone-induced neurotoxicity. To investigate whether the reduction in Prx5 might contribute to rotenone-induced DA neuron degeneration, we knocked down the expression of Prx5 in cultured mesencephalic neurons using specific small interfering RNA prior to rotenone exposure. The results showed that the downregulation of Prx5 made DA neurons more vulnerable to rotenone treatment (Figure 1H).

### 3.2. Prx5 Depletion Sensitizes DA Neuronal Cells to Rotenone-Induced Apoptosis

**Figure 2:**
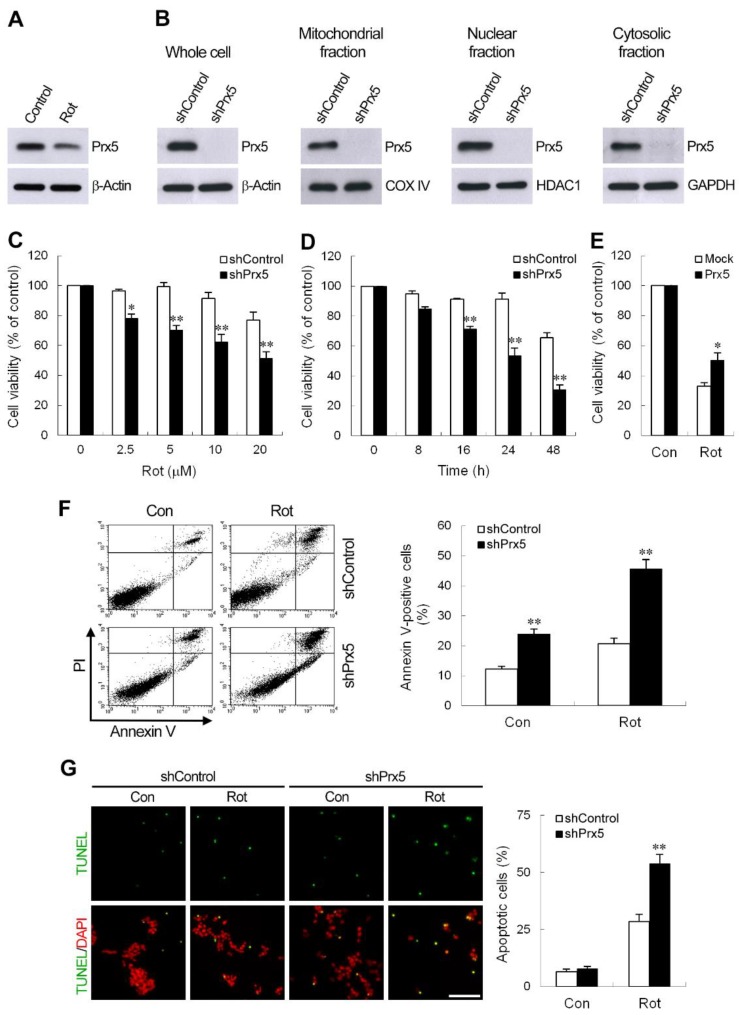
Prx5 depletion resulted in increased sensitivity to rotenone-induced apoptotic cell death. (**A**) Protein expression of Prx5 in whole-cell lysates was confirmed by western blotting in SH-SY5Y cells treated with 10 μM rotenone for 24 h. (**B**) Generation of SH-SY5Y clones silencing Prx5. SH-SY5Y cells were stably transduced with lentivirus expressing shRNA against human Prx5 or scramble control sequence. Whole-cell lysates and subcellular fractionations (cytosolic, mitochondrial, and nuclear fractions) were prepared from control and Prx5-depleted cells. The silencing efficiency of Prx5 was determined by immunoblotting. β-Actin, cytochrome c oxidase IV (COX IV), histone deacetylase 1 (HDAC1), and glyceraldehyde 3 phosphate dehydrogenase (GAPDH) immunoblotting was performed to monitor loading for cell lysates, mitochondrial, nuclear, and cytosol proteins, respectively. (**C**,**D**) Control and Prx5-depleted cells were treated with various concentrations of rotenone for 24 h (**C**) or treated with 10 μM rotenone for the indicated times (**D**). The cell viability was assessed by MTT. Data are presented as mean ± SEM for three independent experiments. * *p* < 0.05; ** *p* < 0.01 compared with respective control shRNA-expressing cells. (**E**) Prx5-depleted cells were transfected for 30 h with a vector containing cDNA of human Prx5 or the empty vector and exposed to 10 μM rotenone. Cell viability was determined at 36 h after rotenone exposure using MTT assay. Data are presented as mean ± SEM for three independent experiments. * *p* < 0.05 compared with rotenone-treated mock vector-transfected cells. (**F**) Cells were treated with 10 μM rotenone for 24 h. Cell apoptosis was assessed using flow cytometry with Annexin V-FITC and propidium iodide (PI) double staining. Representative flow cytometry profiles of three independent experiments are shown (left panel). The apoptotic rate was represented by the percentage of Annexin V-positive cells in the total cell population (right panel). (**G**) Thirty-six hours after treatment with rotenone (10 μM), cells were subjected to TUNEL staining and then examined by fluorescent microscopy. Representative images of TUNEL staining from three independent experiments are shown (left panel). Scale bar = 25 μm. Quantification of apoptotic cells (TUNEL^+^) is shown as a percentage of the total cell number. Data are presented as mean ± SEM for three independent experiments. ** *p* < 0.01 compared with respective control shRNA-expressing cells.

Human DA neuroblastoma SH-SY5Y cells have been extensively used as an in vitro model to explore the cellular and molecular mechanisms underlying the pathogenesis of PD [16,29,30]. To determine whether SH-SY5Y cells could be used as a suitable model for this system, we assessed Prx5 expression in the whole-cell lysates of rotenone-treated SH-SY5Y cells. We found that, as seen in rat mesencephalic DA neurons, the protein level of Prx5 was reduced to about 61.2 ± 2.89% compared to control cells (Figure 2A). Therefore, SH-SY5Y cells were used in the following experiments. To characterize the function of Prx5 in DA neurons responding to rotenone exposure, we knocked down Prx5, using short hairpin interfering RNA (shRNA). In this study, SH-SY5Y cells were infected with lentivirus carrying an shRNA targeted to human Prx5, and stable clones were obtained following puromycin selection. Immunoblot analyses revealed that the amount of Prx5 was greatly reduced in whole-cell lysates (Figure 2B). Subcellular fractionation further confirmed that in mitochondrial, nuclear, and cytosolic fractions, Prx5 levels were depleted (Figure 2B). To assess the effect of Prx5 knockdown toward rotenone neurotoxicity, we treated control and Prx5-depleted cells with increasing concentrations of rotenone for 24 h, or at 10 μM for different periods. As shown in Figure 2C,D, rotenone induced a dose and time-dependent reduction of cell viability. The knockdown of Prx5 made the cells more susceptible to rotenone exposure by significantly increasing rotenone-induced cell death. To further clarify the role of Prx5 in protecting DA neurons from rotenone-induced damage, Prx5 knock-in experiments were carried out. As shown (Figure 2E), knock-in of Prx5 significantly attenuated rotenone neurotoxicity in Prx5-depleted cells. Activation of the mitochondrial apoptotic cascade is thought to cause neuronal death and may participate in neurodegenerative processes [31]. Rotenone has been shown to induce the apoptotic neuronal death [32,33]. We next examined the effect of Prx5 depletion on apoptosis in SH-SY5Y cells. To detect cell apoptosis, SH-SY5Y cells were analyzed by flow cytometry method after staining with Annexin V and PI (Figure 2F). The results revealed that the Annexin V-positive population (early plus late apoptosis) was increased, even in the absence of rotenone, in Prx5-depleted cells compared with that in control cells. The proportion of apoptotic cells induced by rotenone was also higher in Prx5 knockdown cells than in control cells. Similarly, TUNEL analysis indicated that the apoptotic death of cells was induced by increasing the number of TUNEL-positive cells following rotenone treatment (Figure 2G). Compared to rotenone-treated control cells, knockdown of Prx5 significantly increased the number of TUNEL-positive cells. These results indicated that Prx5 depletion sensitized DA cell line SH-SY5Y to rotenone-induced cell death.

### 3.3. Knockdown of Prx5 Exacerbates Mitochondria-Driven Apoptotic Pathway by Rotenone

**Figure 3:**
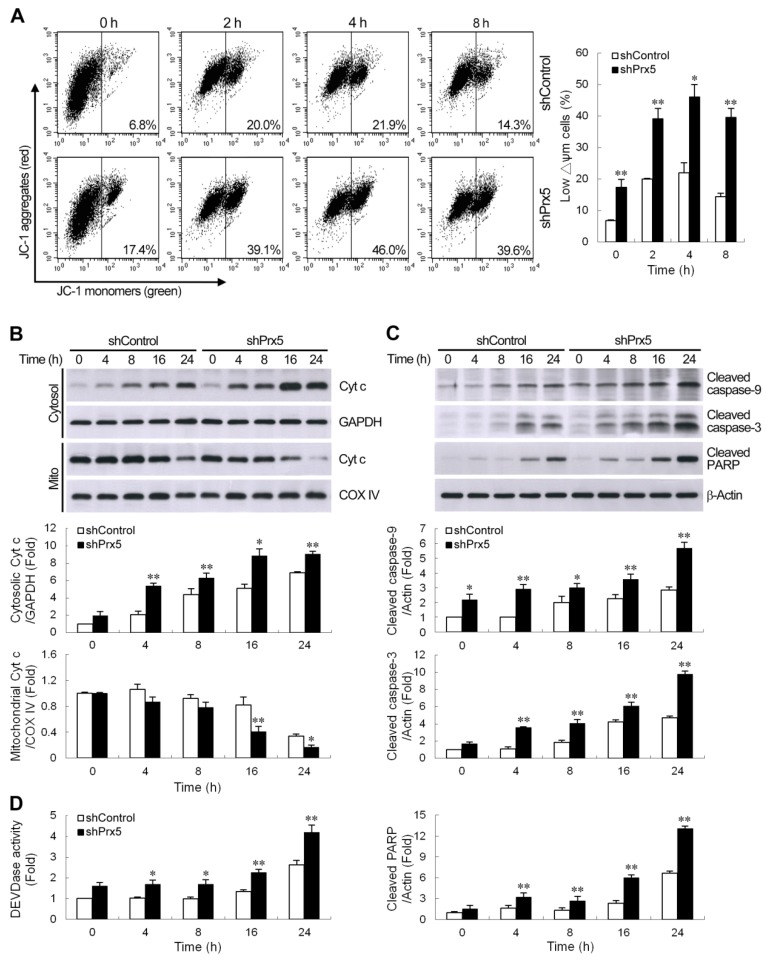
The knockdown of Prx5 exacerbated mitochondria-mediated apoptotic signaling by rotenone. (**A**) Control and Prx5-depleted cells were cultured with rotenone (10 μM) for the indicated times, and changes in mitochondrial membrane potential were then evaluated by staining with JC-1 (5,5′,6,6′-tetrachloro-1,1′,3,3′-tetraethyl-benzimidazolylcarbocyanine iodide). Representative histograms of flow cytometry analysis of JC-1 staining are shown (left panels). The right panel shows the percentage of cells with a low Δψ*m* (increased green fluorescence of the JC-1 monomer). (**B**) Cells were exposed to rotenone (10 μM) for the indicated times. Cytochrome c protein levels were assessed in cytosolic and mitochondrial (Mito) fractions by western blot analysis. Immunoblots for GAPDH and COX IV are shown for normalization of cytosolic and mitochondrial fractions, respectively. (**C**,**D**) After exposure to rotenone, whole-cell lysates were prepared, and the activation of caspases cascade was determined by western blotting (**C**). The graphs show the quantification analysis of cleaved caspase-9, cleaved caspase-3, and cleaved poly (ADP-ribose) polymerase (PARP)/β-actin. Caspase-3-like protease activity (**D**) in cell lysates was also measured by cleavage of the fluorogenic substrate Ac-DEVD-AMC. Caspase-3-like activity from the non-stimulated control shRNA-expressing cells was arbitrarily set at 1 for the calculation of fold. Data are presented as mean ± SEM for 3–4 independent experiments. * *p* < 0.05; ** *p* < 0.01 compared with respective control shRNA-expressing cells.

It is well known that loss of mitochondrial membrane potential (Δψ*m*), increased cytochrome c release, and activation of caspases are involved in the progress of mitochondria-mediated apoptosis. We, therefore, examined the effects of Prx5 depletion on these events. In this study, the potential-sensitive fluorescent probe JC-1 was employed to detect the change in Δψ*m*. In normal mitochondria, JC-1 forms aggregates in mitochondria that emit red fluorescence. If mitochondrial membranes are depolarized, JC-1 exists as green fluorescent monomers in the cytosol. Flow cytometric analysis showed that the percentage of cells with low Δψ*m* was higher in Prx5-depleted cells than that in control cells, even in the absence of rotenone (Figure 3A). Cells with depolarized mitochondria were increased time-dependently after treatment with rotenone, and this effect was enhanced by Prx5 knockdown. Mitochondrial dysfunction could provoke the release of cytochrome c from mitochondria into the cytosol. Immunoblot analysis revealed that rotenone induced mitochondrial release of cytochrome c in SH-SY5Y cells. Furthermore, cultures depleted of Prx5 showed a significant increase in cytochrome c release (Figure 3B). Exposure of cells to rotenone also led to the activation of caspase-9 and caspase-3, the two major caspases associated with the mitochondrial apoptotic pathway, as well as that of the caspase substrate poly (ADP-ribose) polymerase (PARP). Again, Prx5 knockdown augmented the activation of this caspase cascade following rotenone treatment (Figure 3C). Measurement of caspase-3 activity with a specific fluorogenic substrate, DEVD-AMC, further confirmed that the activation of caspase-3 by rotenone was enhanced in Prx5-depleted cells (Figure 3D). These results suggested that Prx5 depletion enhanced rotenone-induced apoptosis of SH-SY5Y cells by increasing the activation of the intrinsic (also termed mitochondrial) apoptotic pathway.

### 3.4. Silencing of Prx5 Enhances the Activation of PUMA-Mediated Mitochondrial Apoptotic Pathway

**Figure 4:**
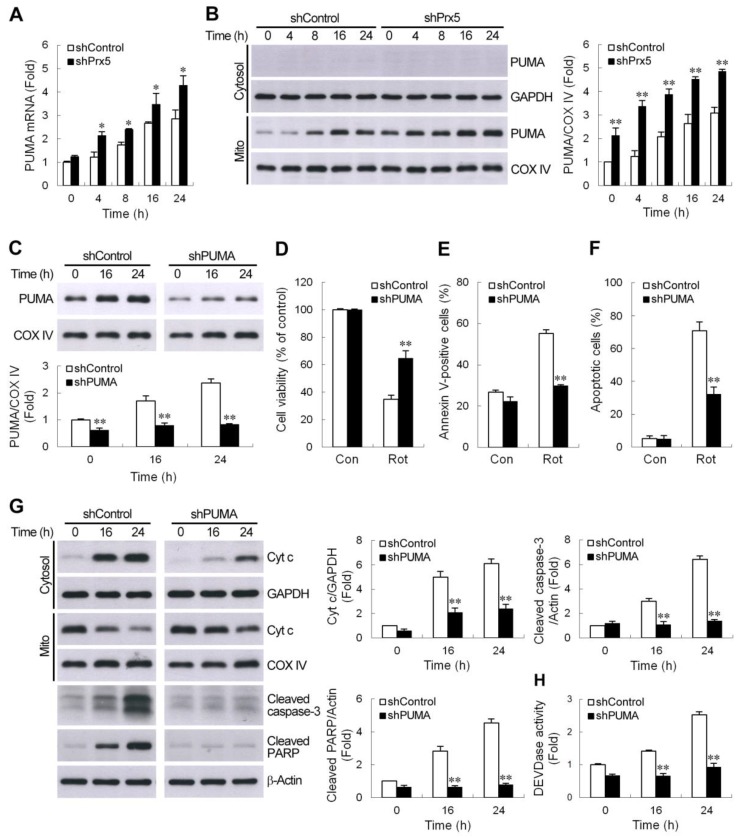
PUMA contributed to Prx5 silencing-enhanced activation of the mitochondrial apoptotic pathway. (**A**,**B**) Prx5 knockdown increased rotenone-induced PUMA expression. Control and Prx5-depleted cells were treated with rotenone (10 μM) for the indicated times. The expression of PUMA mRNA (**A**) was analyzed by real-time RT-PCR. PUMA protein levels (**B**) were assessed in cytosolic and mitochondrial fractions by western blot analysis. GAPDH and COX IV were used as the cytosolic and mitochondrial fraction markers for loading, respectively. Data are presented as mean ± SEM for three independent experiments. * *p* < 0.05; ** *p* < 0.01 compared with respective control shRNA-expressing cells. (**C**–**F**) Downregulation of PUMA in Prx5 knockdown cells increased cell survival and mitigated apoptosis following rotenone treatment. Prx5-depleted cells were infected with lentivirus expressing non-targeted (shControl) or PUMA-targeted shRNA (shPUMA). Stable clones of each group were selected. Expression levels of PUMA protein induced by rotenone in the mitochondrial fractions were analyzed by western blotting (**C**). The blots from both cells were exposed to the same X-film (Supplementary images). Data are presented as mean ± SEM for three independent experiments. ** *p* < 0.01 compared with respective control shRNA-expressing cells. Control and PUMA knockdown cells were exposed to rotenone for 36 h. Cell viability and apoptosis were analyzed by MTT (**D**) and Annexin V-APC (**E**) or TUNEL (**F**) staining, respectively, as described in Figure 2. Data are presented as mean ± SEM for three independent experiments. ** *p* < 0.01 compared with rotenone-treated control shRNA-expressing cells. (**G**,**H**) PUMA was essential for the Prx5 loss-aggravated rotenone-induced mitochondrial apoptotic pathway. Control and PUMA knockdown cells were stimulated with rotenone for the indicated times. Cytosolic, mitochondrial fractions, or whole-cell extracts were subjected to immunoblot analysis with antibodies to cytochrome c, cleaved caspase-3, and cleaved PARP (**G**). The blots from both cells were exposed to the same X-film (Supplementary images). Caspase-3 like activity in cell lysates was also determined with the synthetic substrate (**H**). Data are presented as mean ± SEM for four independent experiments. ** *p* < 0.01 compared with respective control shRNA-expressing cells.

Members of the Bcl-2 family proteins can be activated by a variety of apoptotic stimuli and have been shown to be critical regulators of the intrinsic mitochondria-dependent apoptotic pathways. Furthermore, Bax/Bak activation is known to be modulated by the actions of Bcl-2 homology 3 (BH3)-only Bcl-2 family proteins during apoptosis [34]. Among them, a p53 upregulated modulator of apoptosis (PUMA) is one of the most potent killers [35]. Therefore, we thought to determine whether augmentation of PUMA expression contributes to the worse effect of Prx5 depletion in rotenone-induced cell death. We first examined the expression profiles of PUMA by quantitative real-time PCR. The results showed that treatment with rotenone significantly increased PUMA gene expression in a time-dependent manner in SH-SY5Y cells. The knockdown of Prx5 in SH-SY5Y cells was shown to further enhance the expression of PUMA mRNA (Figure 4A). As illustrated in Figure 4B, rotenone also caused an increased protein level of PUMA, specifically within the mitochondrial fraction. Similar to the mRNA expression profiles, the levels of PUMA protein in Prx5-depleted cells were higher than those in control cells. We next examined the potential role of PUMA in Prx5 depletion-induced deleterious effect on cell survival following rotenone exposure. To address this, we knocked down PUMA (Figure 4C) in Prx5-depleted cells and treated these cells with rotenone. The results showed that PUMA knockdown increased cell viability (Figure 4D) and reduced the extent of apoptosis (Figure 4E,F). Based on the foregoing results, Prx5 depletion enhanced rotenone-induced apoptosis through increasing the activation of the mitochondrial-dependent apoptotic pathway. We further examined the contribution of PUMA in rotenone-induced mitochondrial apoptotic pathways in Prx5-depleted cells. Consistent with the observed protection afforded by PUMA knockdown, cytochrome c release, as well as caspase-3 cascade activation induced by rotenone, was significantly attenuated in PUMA-silenced cells (Figure 4G,H). Together, our data indicated that increased sensitivity of Prx5-depleted cells to rotenone-induced apoptosis was attributed to the elevation of PUMA.

### 3.5. Prx5 Downregulation Strengthens the p53-PUMA-Mediated Apoptotic Pathway

**Figure 5:**
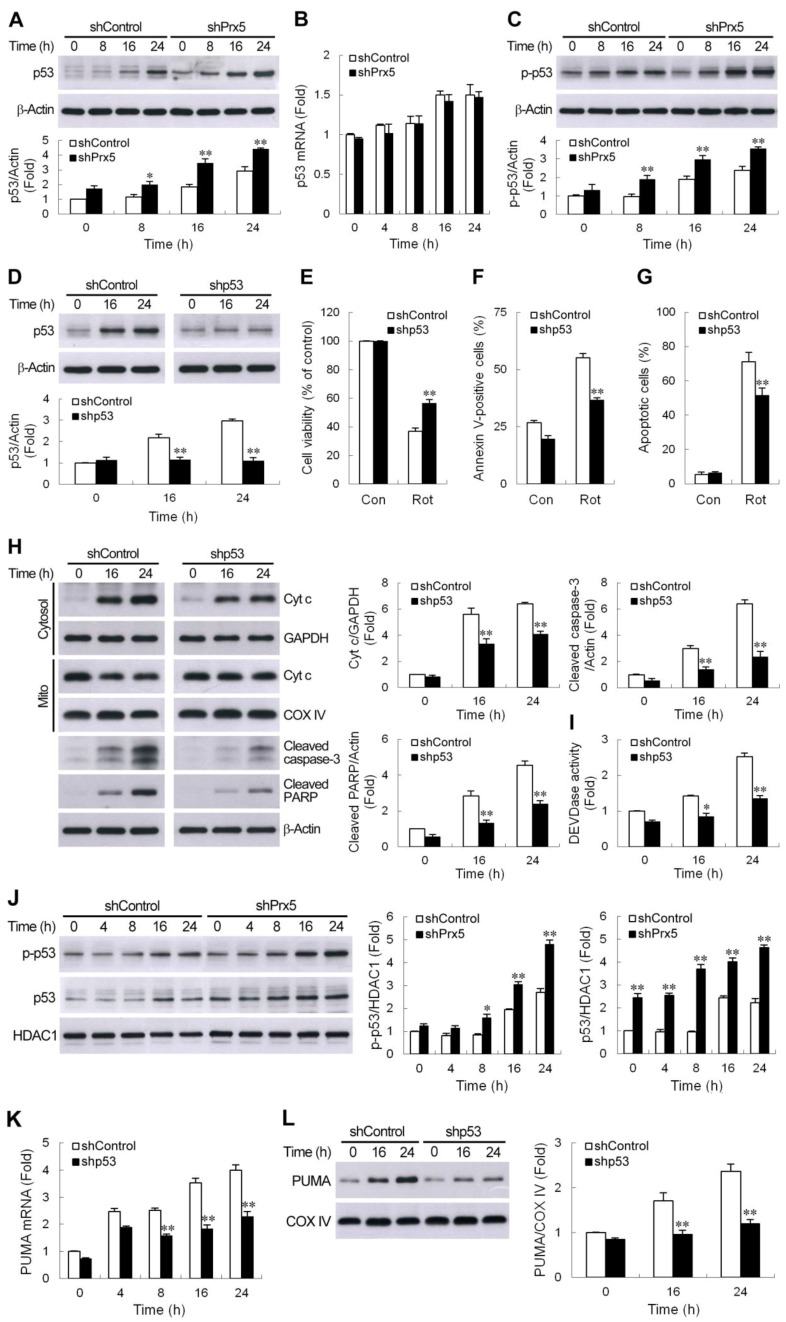
Prx5 downregulation strengthened the PUMA-mediated apoptotic pathway via a p53-dependent mechanism. (**A**–**C**) Loss of Prx5-enhanced rotenone-induced p53 protein accumulation was not dependent on transcriptional activation. Control and Prx5-depleted cells were treated with 10 μM rotenone for the indicated period, the cell lysates were prepared, and immunoblotting was carried out for p53 and p-p53 (Ser15) (**A**,**C**). The induction of p53 mRNA expression was quantified by real-time RT-PCR (**B**). Data are presented as mean ± SEM for three independent experiments. * *p* < 0.05; ** *p* < 0.01 compared with respective control shRNA-expressing cells. (**D**–**G**) The knockdown of p53 in Prx5-silencing cells protected against rotenone-induced neurotoxicity. Prx5-depleted cells were transduced with lentivirus carrying shRNA targeting p53 (shp53) or the scrambled control sequence (shcontrol). Stable clones of each group were selected. Rotenone-induced expression of p53 in the whole-cell lysates was analyzed by western blotting (**D**). The blots from both cells were exposed to the same X-film (Supplementary images). Cell viability and apoptosis caused by rotenone were assessed, as described in Figure 4 (**E–G**). Data are presented as mean ± SEM for three independent experiments. ** *p* < 0.01 compared with respective control shRNA-expressing cells in (**D**). ** *p* < 0.01 compared with rotenone-treated control shRNA-expressing cells in (**E**–**G**). (**H**,**I**) Control and p53 knockdown cells were stimulated with rotenone. At the indicated time points, cytosolic, mitochondrial fractions, or whole-cell extracts were subjected to immunoblot analysis with antibodies, as described in Figure 4G (**H**). The blots from both cells were exposed to the same X-film (Supplementary images). Caspase-3 like activity in cell lysates was also determined with the synthetic substrate (**I**). Data are presented as mean ± SEM for four independent experiments. * *p* < 0.05; ** *p* < 0.01 compared with respective control shRNA-expressing cells. (**J**–**L**) p53 is a key regulator of Prx5 silencing-upregulated PUMA expression. Western blot analysis of p-p53 (Ser 15) and p53 in the nuclear fractions prepared from rotenone-treated control and Prx5-depleted cells (**J**). Control and p53 knockdown cells were treated with rotenone as above. PUMA mRNA induction and protein accumulation were determined by real-time RT-PCR (**K**) and western blotting (**L**), respectively. Data are presented as mean ± SEM for four independent experiments. * *p* < 0.05; ** *p* < 0.01 compared with respective control shRNA-expressing cells.

Several lines of evidence have demonstrated that p53-mediated induction of PUMA is essential for neuronal apoptosis induced by DNA damage [36,37,38] as well as oxidative stress [39]. Accordingly, we next investigated whether a p53-PUMA pathway is involved in rotenone-mediated neuronal death and whether Prx5 depletion enhances p53 expression. As shown in Figure 5A, immunoblot analysis revealed that rotenone induced p53 protein accumulation in SH-SY5Y cells. After exposure to rotenone, the silencing of Prx5 resulted in a larger increase in p53 protein levels compared to control cells. The increased p53 protein levels by rotenone might be due to transcriptional induction of p53 and/or stabilization of the protein owing to post-translational modification. To address this issue, we first examined whether p53 mRNA was increased in rotenone-stimulated cells using real-time RT-PCR. Rotenone treatment slightly induced p53 mRNA expression in SH-SY5Y cells (Figure 5B). However, there was no significant difference in p53 mRNA levels between rotenone-treated control and Prx5-depleted cells, suggesting that the differential induction of p53 was not dependent on gene transcription, and a mechanism in addition to transcription might contribute to p53 augmentation in shPrx5-expressing cells. It is generally believed that post-transcriptional modifications like phosphorylation play a role in stabilizing p53 protein. Murine double minute 2 (MDM2) inhibits p53 accumulation by targeting it for ubiquitination and proteasomal degradation [40]. Phosphorylation of p53 at Ser15 impairs the ability of MDM2 to bind p53 and leads to the accumulation of p53 [41]. Our data showed that rotenone treatment resulted in an increase in phosphorylation of the serine 15 residue of p53 in cell lysates extracted from both cells (Figure 5C). Similarly, a higher level of this phosphorylated p53 was seen in Prx5-depleted cells compared to control cells. Since we observed an increased stabilization of p53 in the rotenone-treated Prx5-depleted cells, we wanted to determine if p53 plays a role in rotenone-induced neuronal cell death exacerbated by Prx5 loss. We knocked down p53 (Figure 5D) using a lentiviral expression system containing shRNA to p53 and analyzed cell viability and apoptosis in Prx5-depleted cells following exposure to rotenone. The results showed that cell death and apoptosis caused by rotenone in Prx5-depleted cells were significantly prevented by p53 shRNA (Figure 5E–G). Consistent with the significant decrease in apoptotic cells, the cytochrome c release and caspase-3 activation were also found to be reduced in p53 silencing cells (Figure 5H,I).

P53 phosphorylation improves its stability and promotes the recruitment of transcriptional coactivators. After p53 stabilization, p53 accumulates in the nucleus to directly regulate the expression of pro-apoptotic genes. PUMA has been shown to be a critical downstream transcriptional target in p53-mediated neuronal cell death. Accordingly, we investigated whether p53 modulates rotenone-caused PUMA induction. We first examined the nuclear accumulation of p53 by western blotting. Similar results were obtained, as seen in Figure 5A,C, with Prx5 knockdown, which also increased rotenone-induced phosphorylation of p53 in the nucleus (Figure 5J), and which was accompanied by an increase in its total protein level. Furthermore, the downregulation of p53 in Prx5-silenced cells was shown to significantly reduce the mRNA and protein levels of PUMA (Figure 5K,L). Together, our findings suggested that rotenone triggered PUMA induction and neuronal cell death via, at least partially, a p53-dependent mechanism, and that loss of Prx5 could strengthen this p53-PUMA-mediated apoptotic pathway.

### 3.6. Prx5 Loss Augments DNA Damage-Triggered ATM/p53 Signaling

**Figure 6:**
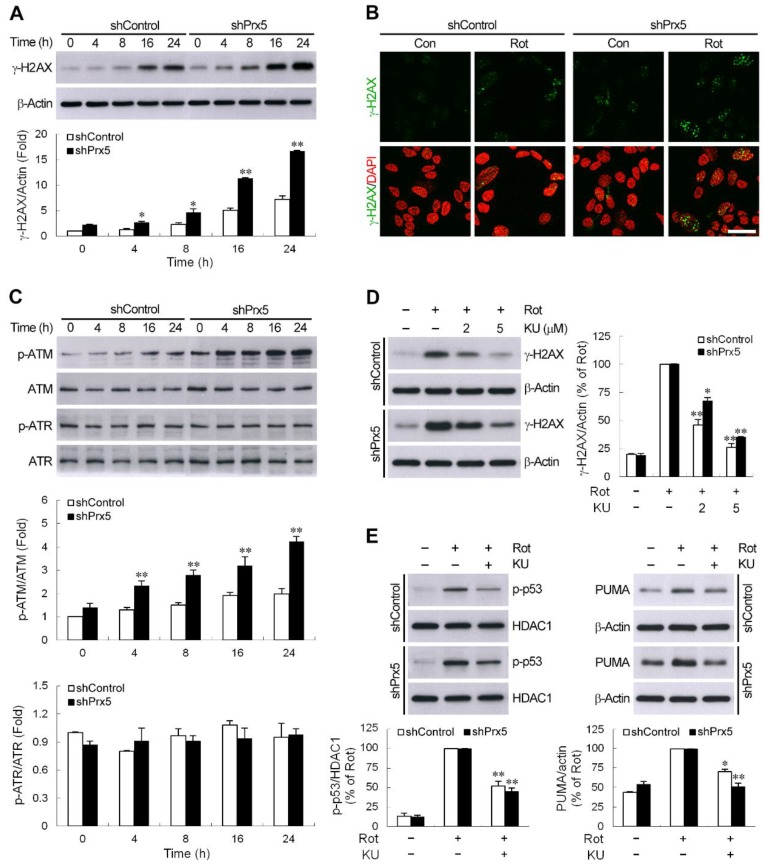
DNA damage-induced ATM/p53 signaling was augmented by Prx5 loss. (**A**) Control and Prx5-depleted cells were exposed to rotenone (10 μM) for the indicated times. The level of serine 139-phosphorylated H2AX (γ-H2AX) was detected by western blotting, with β-actin used as a loading control. Data are presented as mean ± SEM for four independent experiments. * *p* < 0.05; ** *p* < 0.01 compared with respective control shRNA-expressing cells. (**B**) Immunofluorescence staining of cells showing γ-H2AX production (green) after exposure to rotenone for 24 h. Nuclei were counterstained with DAPI. Images were captured using a fluorescence microscope. Scale bar = 25 μm. (**C**) After incubation with rotenone as above, the cell lysates were analyzed by western blotting using antibodies specific to phosphorylated ATM (Ser1981) and ATR (Ser428), and their total forms were used as a loading control. Data are presented as mean ± SEM for four independent experiments. ** *p* < 0.01 compared with respective control shRNA-expressing cells. (**D**,**E**) Control and Prx5-depleted cells were pretreated with 2 and 5 μM (**D**) or 5 μM (**E**) KU-55933, a selective ATM inhibitor, for 1 h followed by exposure to rotenone for another 16 h. Western analysis was used to determine γ-H2AX (**D**) and p-p53 and PUMA (**E**) proteins in whole-cell lysates or nuclear fractions. The blots from both cells were exposed to the same X-film (Supplementary images). Data are presented as mean ± SEM for four independent experiments. * *p* < 0.05; ** *p* < 0.01 compared with respective rotenone alone.

One of the most potent activators of p53 is DNA damage. To confirm the occurrence of DNA damage in rotenone-treated cells, we assessed at different time points the phosphorylation of histone H2AX (γ-H2AX) at Ser139, a known marker of DNA double-strand breaks (DSBs) [42,43], by Western blotting. As shown in Figure 6A, the constitutive level of γ-H2AX was higher in Prx5-depleted cells compared with that in control cells. Following treatment with rotenone, the expression of γ-H2AX in both cells was increased with time, and the extent of γ-H2AX accumulation was more pronounced by Prx5 knockdown. Moreover, the differential induction of γ-H2AX was also observed by fluorescent staining (Figure 6B). These results indicated that the downregulation of Prx5 caused more severe DNA damage following rotenone exposure. H2AX is phosphorylated by kinases, such as ataxia telangiectasia mutated (ATM) and ataxia telangiectasia and Rad3-related (ATR) [43,44], both kinases are also known to induce phosphorylation of p53 at serine 15 [41]. Activation of ATM or ATR by phosphorylation occurs in response to DNA damage [45,46]. To test whether the activation of ATM or ATR is induced by rotenone and the effect of Prx5 on this rotenone’s action, immunoblot assays were performed to determine the phosphorylation state of these kinases. The results showed that the levels of phosphorylated ATM rose in rotenone-treated cells, whereas phosphorylated ATR levels remained unaffected (Figure 6C). Consistent with the differential induction of γ-H2AX, Prx5 silencing also significantly elevated the ATM phosphorylated levels. To examine the participation of ATM on the DNA damage response following the rotenone treatment, we analyzed the induction of γH2AX in the presence of a specific inhibitor of ATM, KU-55933 [44]. We found that inhibition of ATM activity by KU-55933 limited rotenone-induced γH2AX accumulation (Figure 6D). Furthermore, consistent with H2AX and p53 (Ser15) phosphorylation being dependent on ATM, KU-55933 also suppressed the increase in phosphorylation of p53 (Figure 6E). At the same time, the expression of PUMA was also shown to be significantly attenuated by this inhibitor (Figure 6E). These results indicated that prx5 might prevent ATM/p53-mediated apoptosis cascade via the mitigation of DNA damage induced by rotenone.

### 3.7. ROS Does Not Contribute to Prx5 Depletion-Increased γ-H2AX Induction by Rotenone

**Figure 7:**
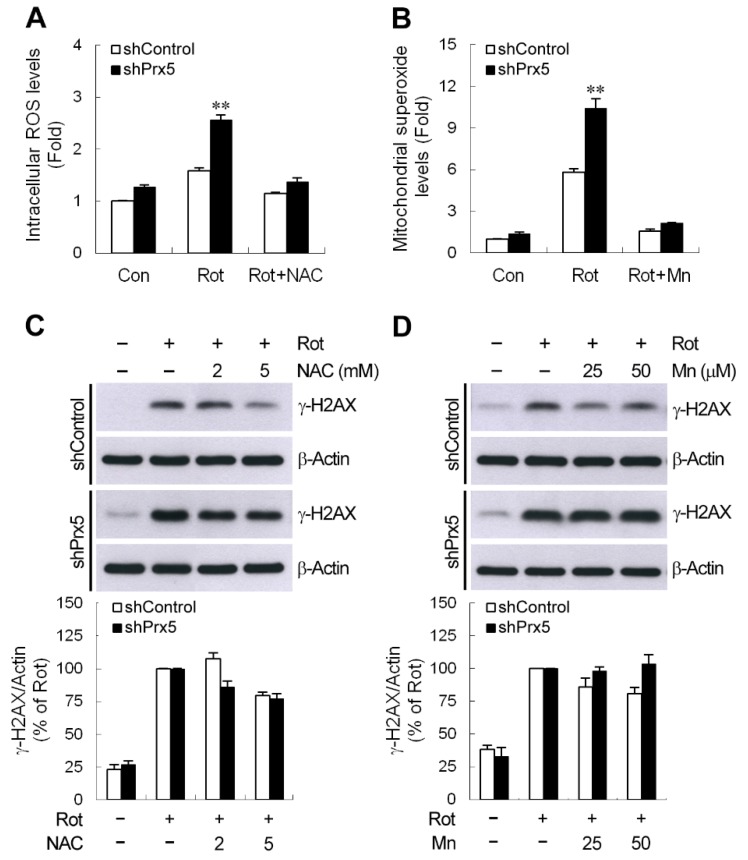

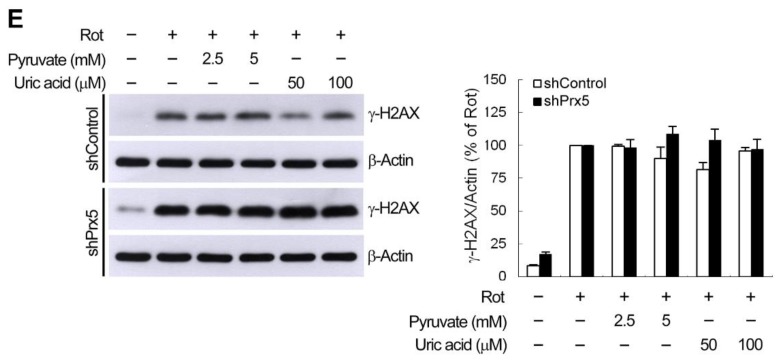
Prx5 depletion-increased γ-H2AX accumulation was not attributed to reactive oxygen species (ROS) production. (**A**,**B**) Control and Prx5-depleted cells were incubated with 10 μM rotenone for 24 h (**A**) or 4 h (**B**) in the absence or presence of NAC (*N*-acetyl-l-cysteine) (5 mM) (**A**) or manganese (III)-tetrakis (4-benzoic acid) porphyrin (MnTBAP) (Mn, 50 μM) (**B**). Intracellular ROS and mitochondrial superoxide levels were measured using carboxy-H_2_DCFDA and MitoSOX, respectively, by performing flow cytometry. The mean fluorescence intensity was used to represent relative ROS and superoxide levels, and the non-stimulated control shRNA-expressing cells were arbitrarily set at 1 for the calculation of fold. Data are presented as mean ± SEM for three independent experiments. ** *p* < 0.01 compared with rotenone-treated control shRNA-expressing cells. (**C**–**E**) Cells were pretreated with the indicated concentrations of NAC (**C**), MnTBAP (**D**), and sodium pyruvate or uric acid (**E**) for 1 h prior to the exposure of rotenone for another 16 h. Accumulation of γ-H2AX was determined by immunoblotting. The blots from both cells were exposed to the same X-film (Supplementary images). Data are presented as mean ± SEM for three independent experiments.

ROS are the most common mediator of DNA damage. Prx5 protects neuronal cells against toxin-induced death through the elimination of subcellular ROS [16,17,18]. Therefore, we evaluated the effect of Prx5 depletion on the rotenone-caused increase in intracellular or mitochondrial ROS. Our results showed that the increased levels of intracellular ROS and mitochondrial superoxide were higher in Prx5-depleted cells following rotenone exposure (Figure 7A,B). Furthermore, cells treated with *N*-acetyl-l-cysteine (NAC) or manganese (III)-tetrakis (4-benzoic acid) porphyrin (MnTBAP), intracellular and mitochondrial ROS scavengers, respectively, were shown to abolish rotenone-induced ROS production. To further determine whether knockdown of Prx5 aggravates rotenone-induced DNA damage by increasing the level of ROS, γH2AX accumulation by rotenone was examined in the presence of ROS scavengers. We found that, unexpectedly, rotenone-induced γH2AX expression was either slightly reduced or unaffected following both NAC and MnTBAP treatment (Figure 7C,D). Regarding the ability of Prx5 to scavenge H_2_O_2_ and ONOO^−^, the targeted scavenger was used to determine whether rotenone-caused γ-H2AX DNA damage could be rescued in Prx5-depleted cells. As shown in Figure 7E, neither sodium pyruvate (H_2_O_2_ scavenger) nor uric acid (ONOO^−^ scavenger) was able to block the accumulation of γH2AX. Our findings suggested that the worse effect of Prx5 loss on rotenone-induced DSBs-mediated DNA damage might be not associated with an increase in ROS production.

## 4. Discussion

The differential expression patterns of six Prx isoforms are observed in distinct brain regions and different cell types. Among them, Prx2, 3, 4, and 5 are expressed in neurons [47,48]. Moreover, the altered expression of Prx subtypes has been reported in a patient’s brain or experimental models of neurodegenerative disorders [18,20,21,49]. Proteome analysis reveals upregulation of Prx1 and Prx6 in experimental models of familial amyotrophic lateral sclerosis [50,51]. Using both in vivo and in vitro models of Alzheimer’s disease, two independent studies indicated that Prx5 rose in these models [17,18]. These observations suggest that changes in the expression of Prxs may be implicated in neurodegenerative pathologies. However, little information is available on the expression of Prx1–6 in PD. Given the downregulation of Prx5, the strongest among the six Prx subtypes in rotenone-treated DA neurons in vitro and in vivo, it is suggested that the decrease of Prx5 might make DA neurons more vulnerable to rotenone exposure. Indeed, the knockdown of Prx5 in primary cultures of mesencephalic neurons or DA cell line SH-SY5Y enhanced the rotenone-induced DA neuronal death. Prx5 is located in various subcellular organelles, and the overexpression of subcellular organelle-specific Prx5 has been shown to protect cells against toxin-induced damage [16,52,53]. The present results suggested that Prx5 might exert a neuroprotective effect in PD models where mitochondrial toxins, such as rotenone or MPP^+^, are administered [16]. A previous study reported that microinjection of ibotenic acid into the rat hippocampus resulted in a decrease of Prx3 mRNA and protein in hippocampal neurons [54]. Furthermore, Prx family proteins, such as Prx1, Prx2, Prx4, and Prx5, have been described to be secreted from cells via exosomal release or an unknown release pathway in a number of oxidative stress/inflammatory models or cancer cells [55,56,57,58]. The mechanism, through either reducing mRNA expression or enhancing the release of protein from cells, which underlies rotenone challenge-induced downregulation of intracellular Prx5 in DA neurons, merits further exploration.

A multitude of studies implicates that apoptosis contributes to the neuronal degeneration that occurs in certain neurodegenerative disorders [31,59]. Although a spectrum of cell death co-exists in PD [59], apoptosis rather than necrosis is considered as the principal contributor for neurodegeneration in PD [60]. It has been widely assumed that deficiency of mitochondrial complex I is associated with the pathogenesis of neurodegenerative disorders. Moreover, SN DA neurodegeneration linked to complex I dysfunction is, at least in part, attributed to the activation of mitochondria-dependent apoptotic pathways [27,61]. Prxs are known to exert anti-apoptotic effects. Overexpression of Prx1, Prx2, or Prx6 significantly protects neurons from neurotoxins-induced apoptosis [23,28,62]. Herein, we demonstrated that depletion of Prx5 exacerbated the loss of Δψ*m* and increased cytochrome c release, followed by activation of the caspase-9- and caspase-3-dependent intrinsic apoptotic pathway after rotenone treatment, suggesting that Prx5 had anti-apoptotic effects in this cellular model of PD. This is consistent with other studies describing that Prx5 might be a potential inhibitor of MPP^+^- or amyloid-β oligomer-induced neurodegeneration by preventing the activation of the mitochondria-mediated apoptotic pathway [16,17,18]. Our experiments further supported the proposal that Prx5 plays a critical role in mitochondrial complex I inhibition-mediated DA neuronal apoptotic signaling.

Mitochondria-mediated apoptotic cell death is modulated by the Bcl-2 protein family comprising anti-apoptotic and pro-apoptotic members. The BH3-only proteins are thought to activate the multidomain pro-apoptotic proteins, such as Bax and/or Bak, to facilitate cytochrome c release from mitochondria, thereby triggering the apoptotic cascade [63,64]. In particular, PUMA is believed to be one of the most powerful killers among the BH3-only proteins [35]. There is substantial evidence indicating PUMA as a potent mediator for neuronal cell death in certain neurodegenerative diseases, including PD [65,66]. Indeed, elevated levels of PUMA have been reported in experimental models of PD in vitro and in vivo, and loss of PUMA prevents DA neuronal death induced by MPP^+^ or 6-OHDA [65,66,67,68]. Consistent with a previous report demonstrating upregulated PUMA is found in a rotenone rat model of PD [69], we detected increased levels of PUMA in SH-SY5Y cells in response to rotenone. Moreover, rotenone-induced PUMA expression was further enhanced in Prx5-knockdown cells, suggesting that loss of Prx5 might intensify PUMA expression, which in turn aggravated apoptotic cell death following exposure to rotenone. As expected, the downregulation of PUMA alleviated Prx5 knockdown-increased rotenone-induced neuronal apoptosis. These findings suggested that the harmful effect of Prx5 silencing on DA neuronal survival was mediated through the PUMA-triggered mitochondrial apoptotic pathway.

The tumor suppressor protein p53 triggers a variety of pro-apoptotic processes, such as the expression of genes associated with apoptosis and pro-apoptotic members of the Bcl2 family [70]. PUMA, a pro-apoptotic p53-target gene, has been shown to be a crucial mediator of p53-initiated apoptotic signaling in several models of neuronal death [36,37,38,39]. Phosphorylation of the p53 at Ser15 disrupts the interaction of p53 with MDM2 protein, promoting both the accumulation and functional activation of p53 in response to DNA damage [41]. The present study demonstrated that rotenone stabilized p53 protein by phosphorylation of p53 on Ser15. Our data were consistent with recent reports showing that p53 is upregulated in rotenone-induced cellular and rat models of PD [71,72]. We also found that rotenone-stimulated p53 induction was enhanced after Prx5 downregulation. Moreover, the knockdown of p53 was shown to ameliorate rotenone-caused neuronal apoptosis in Prx5-depleted cells. Our findings suggested that augmentation of p53 might provide a part of the contribution in the deleterious effect of Prx5 knockdown on the neuronal survival following rotenone exposure. P53-dependent transcriptional activation was manifested as increased PUMA mRNA and protein. Using an RNA interference approach, we further demonstrated that Prx5 depletion-enhanced rotenone-induced PUMA expression was associated with, at least in part, increased p53 activation. Previous studies have reported that the degeneration of DA neurons induced by toxins thought to be relevant to PD is associated with increased levels of p53 and its downstream target PUMA [65,67,68]. Moreover, DA neurons in dopamine transporter-p53 knockout mice lacking DA-specific p53 gene have been shown to be resistant to MPTP [66]. Interestingly, increased phosphorylated p53 has been reported in the SN of postmortem PD brains [73]. Taken together, our data suggested that Prx5 might block the p53-PUMA pathway, ultimately leading to preventing the rotenone-induced activation of mitochondria-mediated apoptosis in DA neurons.

The DNA damage is thought to be a widespread initiator of neuronal apoptosis, and accumulation of damaged DNA has been found in pathological brains, such as in neurodegenerative diseases [5,30,74]. P53 and its downstream signaling pathways are activated in response to DNA damage and play critical roles in DNA damage-induced apoptosis in neurons [37,75,76]. Herein, we found that the downregulation of Prx5 further enhanced the rotenone-induced induction and functional activation of p53, which could be related to the extent of DNA damage. During the process of DNA damage, histone H2AX phosphorylation (γ-H2AX) is associated to DNA DSBs. Occasionally, it can also occur upon the formation of other types of lesions [42,44]. Therefore, the γ-H2AX can serve as a biomarker for genomic DNA damage [42,43]. In the current study, we found that the induction of γ-H2AX was increased in response to rotenone. This was in agreement with previous studies reporting that rotenone induces a γ-H2AX increase in PC12 cells and MN9D DA neuronal cells [77,78]. Moreover, in parallel with p53 activation, the knockdown of Prx5 was shown to elicit a more pronounced accumulation of γ-H2AX following rotenone treatment. In line with our results, the overexpression of Prx5 in the nucleus has been shown to decrease DNA damage (single-strand breaks induction) caused by H_2_O_2_ and *tert*-butyl- hydroperoxide [52], and downregulating the gene provokes the formation of etoposide-induced DNA DSBs [15]. In addition, Kropotov et al. [79] reported that the constitutively expressed Prx5 gene was essential for the genome defense against spontaneous oxidative lesions (8-oxoguanine formation) in DNA. Our findings further highlighted the importance of Prx5 in a decrease of DSBs-evoked DNA damage in a rotenone-induced cellular model of PD. Autophosphorylation of ATM (Ser1981) is important in ATM-mediated signaling during DNA damage [45]. H2AX is phosphorylated on its Ser139 site by ATM in response to DNA DSBs induced by some DNA damaging agents [43]. Furthermore, ATM is required for the phosphorylation of p53 at Ser15 [41]. Pharmacological inactivation of ATM was shown to attenuate rotenone-induced γ-H2AX accumulation, p53 activation, and PUMA expression. Accordingly, ATM appeared to act upstream of p53 signaling triggered by DNA damage to induce cell death in response to rotenone, and this ATM/p53 signaling was aggravated by the loss of Prx5.

DNA damage is known to be associated with ROS in neurological diseases, including PD [6]. Unexpectedly, our results showed that Prx5 silencing-evoked more severe DNA damage manifested as the γ-H2AX expression was not attributed to the higher levels of ROS. It has been shown that ROS, including H_2_O_2_, commonly generate single-strand DNA breaks (SSBs), abasic sites, and oxidized bases but hardly cause DNA DSBs directly [80]. Katsube et al. [44] demonstrated that activation of ATR, rather than DNA DSBs-activated ATM, contributed to H_2_O_2_-caused γ-H2AX formation. However, ATR activation was not seen in our rotenone treatment systems. Furthermore, a previous study revealed that Prx5-repressed etoposide-induced γ-H2AX accumulation was not related to its antioxidant activity but depended on another functional activity of this protein [15]. These observations might, at least partially, explain why blocking ROS production cannot alleviate Prx5 depletion-mediated DSB-resulting DNA damage caused by rotenone. Further studies are necessary to elucidate the underlying mechanism by which Prx5 prevents rotenone-induced DNA DSBs.

## 5. Conclusions

The present data indicated that the accumulation of γ-H2AX in this rotenone-treated DA neuronal model placed DNA damage as an activator of an ATM/p53/PUMA-regulated apoptotic cascade. Furthermore, we provided the first demonstration of a relationship between Prxs and p53-PUMA signaling and showed that Prx5 modulated p53 activation by regulating rotenone-induced DNA damage. Because rotenone exposure results in the decrease of Prx5 expression in DA neurons in vitro and in vivo, these findings underscored the importance of Prx5 for prevention from rotenone-induced DA neurodegenerative processes.

## Supplementary Materials

The following are available online at https://www.mdpi.com/2073-4409/9/1/22/s1, Supplementary images: The full western blots shown in Figure 4C,G; Figure 5D,H; Figure 6D,E; Figure 7C,D.
