# Supplementary material for: Peroxiredoxin 5 Silencing Sensitizes Dopaminergic Neuronal Cells to Rotenone via DNA Damage-Triggered ATM/p53/PUMA Signaling-Mediated Apoptosis
Source: Cells. 2019 Dec 19;9(1):22. doi: 10.3390/cells9010022 (PMC7016837; doi:10.3390/cells9010022)

Fig. 4C

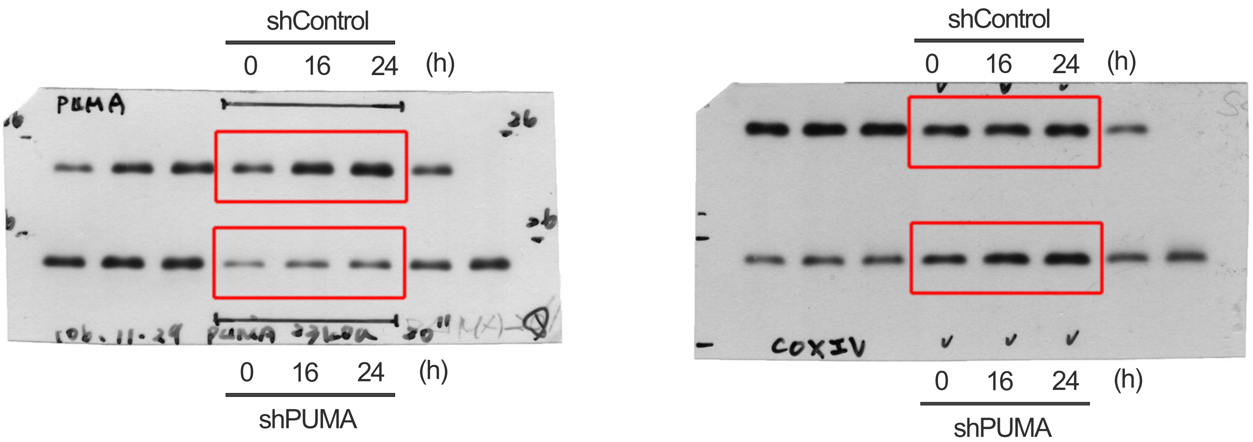
shControl
0 16 24 (h)
PUMA
shPUMA
COX IV

Fig 4G

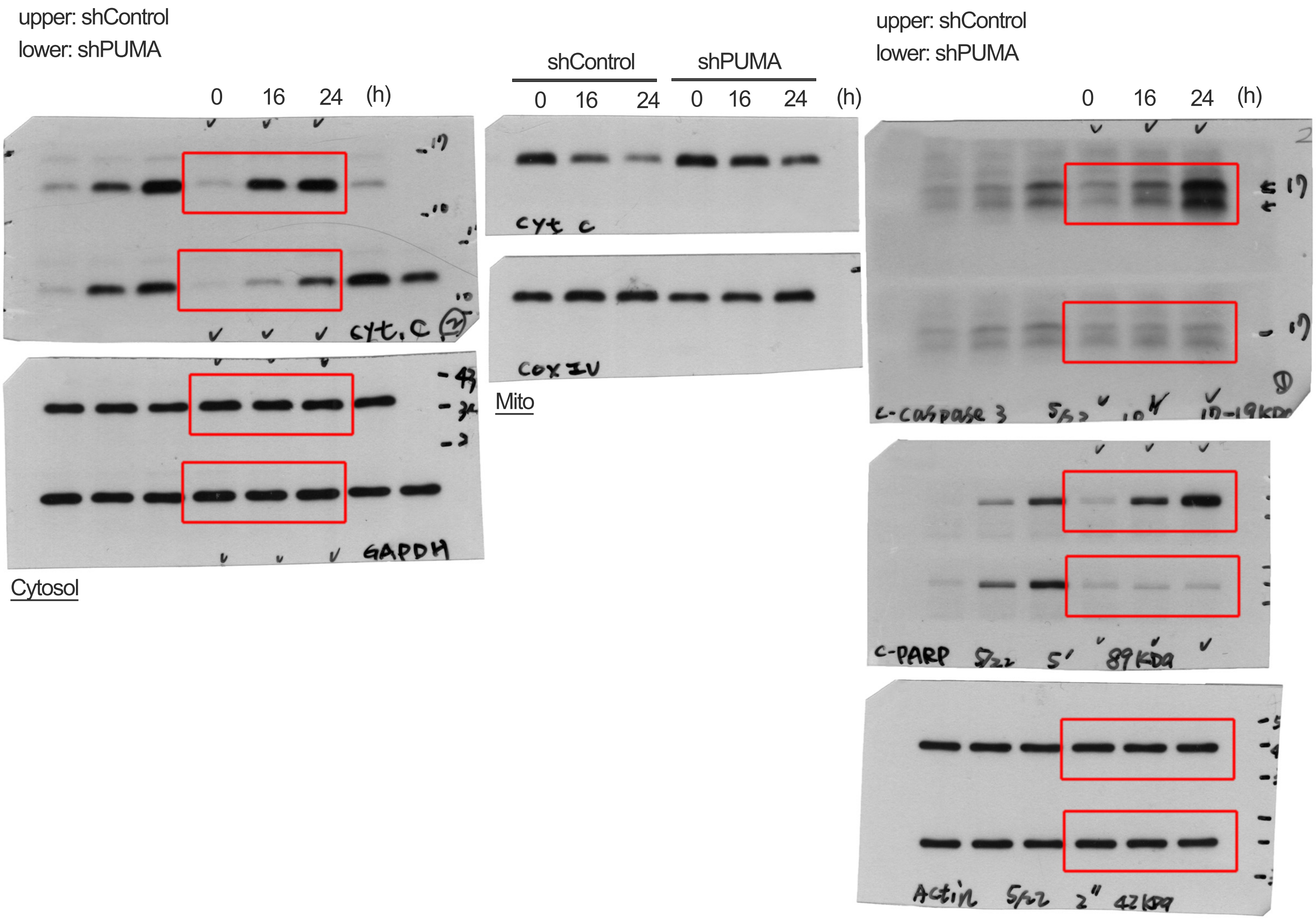
upper: shControl
lower: shPUMA
0 16 24 (h)
cyt. c
GAPDH
Cytosol
shControl
shPUMA
0 16 24 0 16 24 (h)
cyt c
COX IV
Mito
c-caspase 3
c-PARP
Actin

Fig. 5D

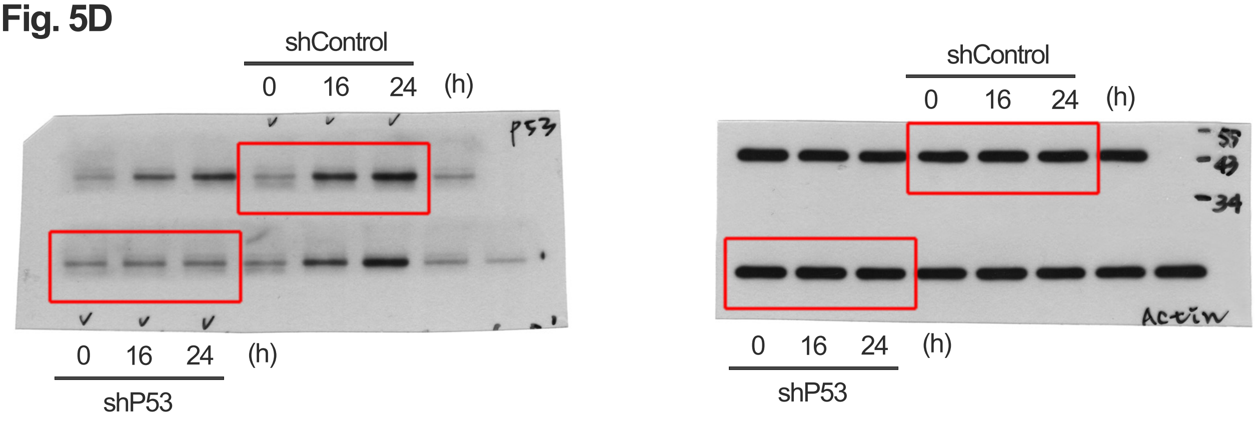
shControl
0 16 24 (h)
p53
0 16 24 (h)
shP53
shControl
0 16 24 (h)
Actin
0 16 24 (h)
shP53

Fig. 5H

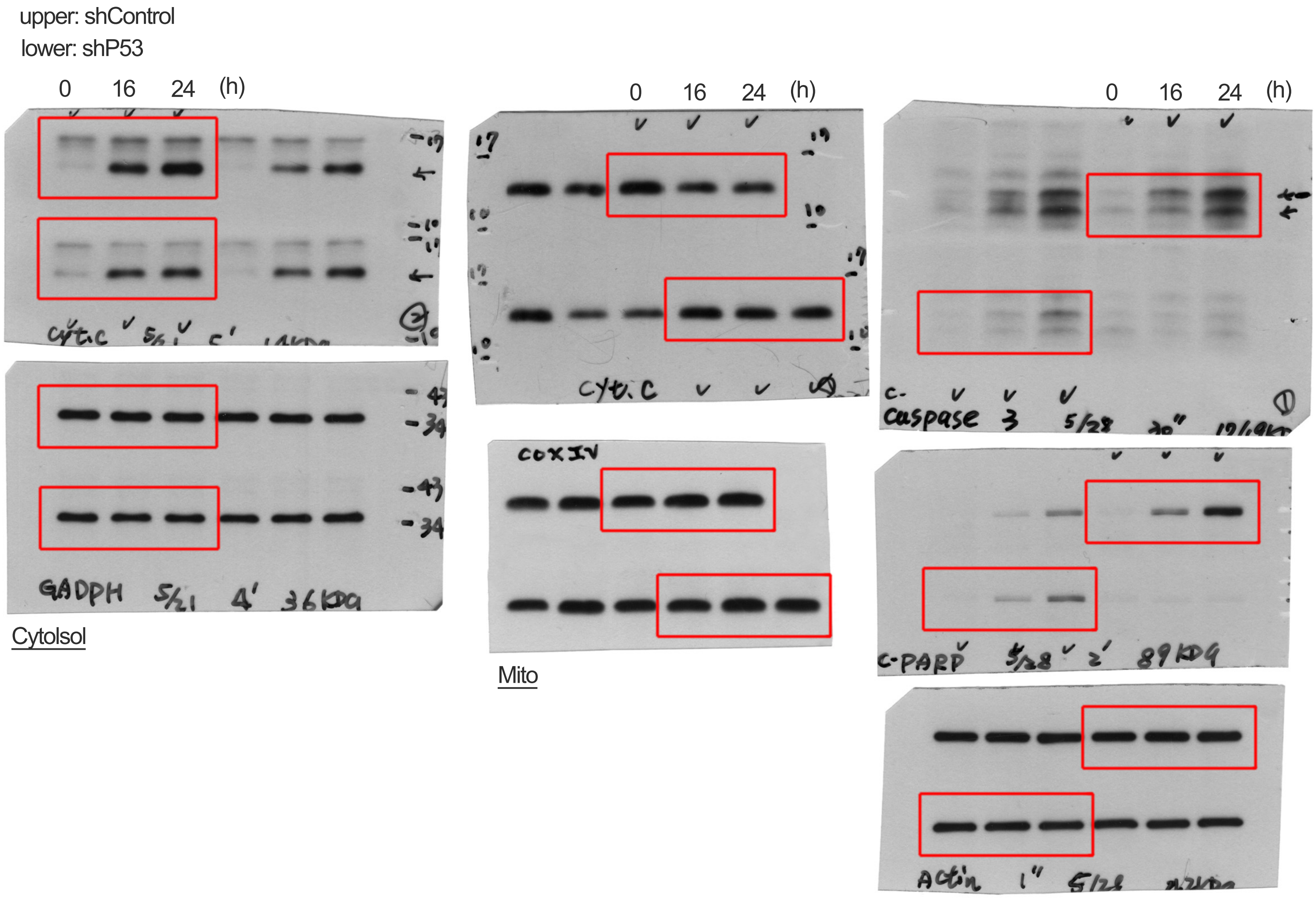
upper: shControl
lower: shP53
0 16 24 (h)
0 16 24 (h)
0 16 24 (h)
Cyt.c
GADPH 5/21 4' 36KDa
Cytolsol
COX IV
Cyt.C
Mito
c-caspase 3 5/28
c-PARP 5/28 2' 89KDa
Actin 1" 5/28

Fig. 6D

upper: shPrx5
lower: shControl

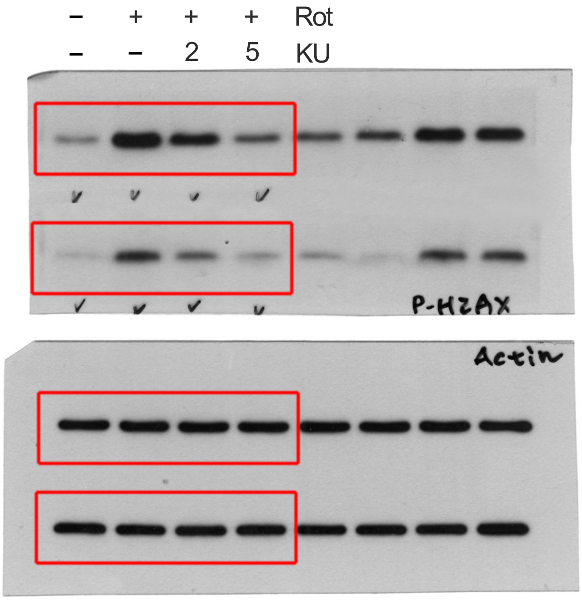

Fig. 6E

upper: shPrx5
lower: shControl

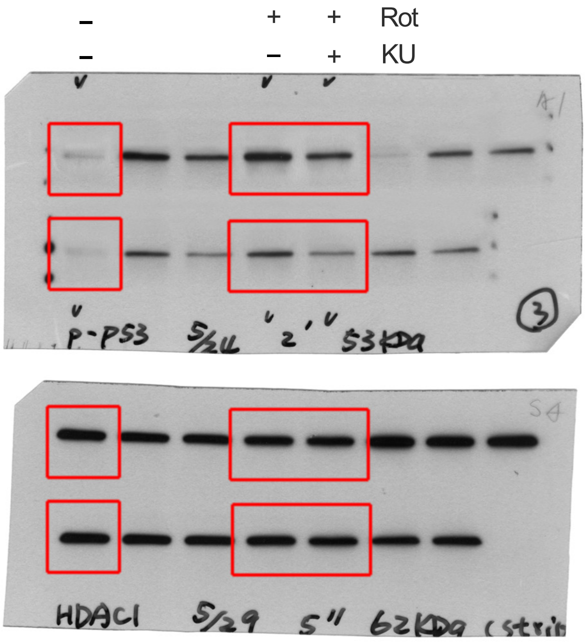

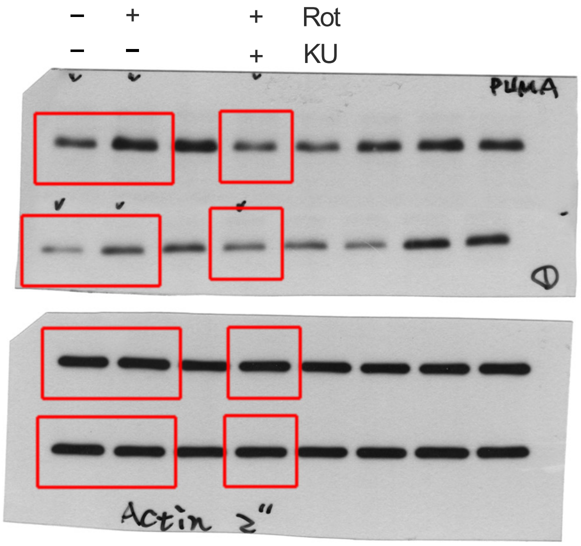

Fig. 7C

upper: shPrx5
lower: shControl

–	+	+	+	Rot
–	–	2	5	Nac

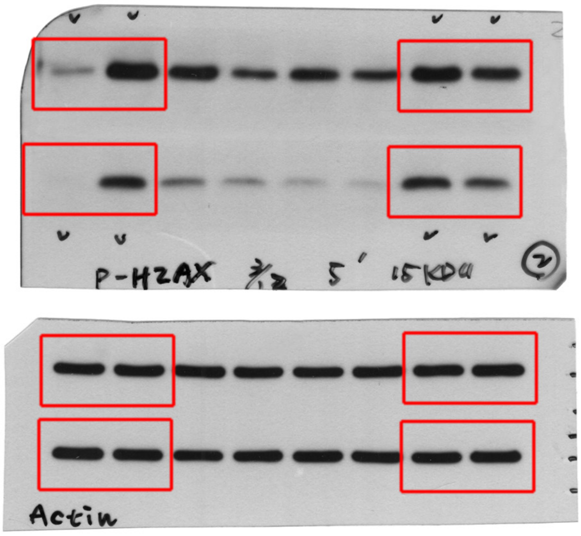

Fig. 7D

upper: shPrx5
lower: shControl

–	+	+	+	Rot
–	–	25	50	Mn

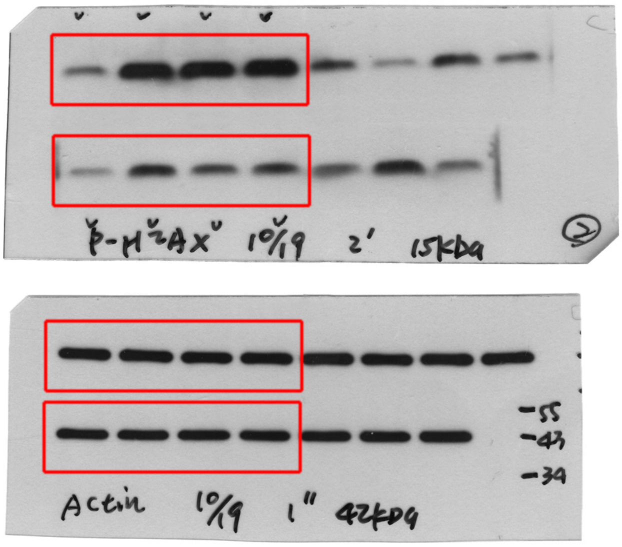

Supplement: Supplementary file 1 [file cells-09-00022-s001.pdf]
